# Trends in Global Vegetation Activity and Climatic Drivers Indicate a Decoupled Response to Climate Change

**DOI:** 10.1371/journal.pone.0138013

**Published:** 2015-10-14

**Authors:** Antonius G. T. Schut, Eva Ivits, Jacob G. Conijn, Ben ten Brink, Rasmus Fensholt

**Affiliations:** 1 Plant Production Systems Group, Wageningen University, Droevendaalsesteeg 1, 6708 PB Wageningen, The Netherlands; 2 Joint Research Centre, Via Enrico Fermi 2749, I - 21027 Ispra, Italy; 3 PBL Assessment Agency for the Environment, Antonie van Leeuwenhoeklaan 9, 3721 MA Bilthoven, The Netherlands; 4 Plant Research International, Wageningen UR, Droevendaalsesteeg 1, 6708 PB Wageningen, The Netherlands; 5 Section of Geography, Department of Geosciences and Natural Resource Management, Faculty of Science, University of Copenhagen, Oster Voldgade 10, 1350 Copenhagen, Denmark; University of California Davis, UNITED STATES

## Abstract

Detailed understanding of a possible decoupling between climatic drivers of plant productivity and the response of ecosystems vegetation is required. We compared trends in six NDVI metrics (1982–2010) derived from the GIMMS3g dataset with modelled biomass productivity and assessed uncertainty in trend estimates. Annual total biomass weight (TBW) was calculated with the LINPAC model. Trends were determined using a simple linear regression, a Thiel-Sen medium slope and a piecewise regression (PWR) with two segments. Values of NDVI metrics were related to Net Primary Production (MODIS-NPP) and TBW per biome and land-use type. The simple linear and Thiel-Sen trends did not differ much whereas PWR increased the fraction of explained variation, depending on the NDVI metric considered. A positive trend in TBW indicating more favorable climatic conditions was found for 24% of pixels on land, and for 5% a negative trend. A decoupled trend, indicating positive TBW trends and monotonic negative or segmented and negative NDVI trends, was observed for 17–36% of all productive areas depending on the NDVI metric used. For only 1–2% of all pixels in productive areas, a diverging and greening trend was found despite a strong negative trend in TBW. The choice of NDVI metric used strongly affected outcomes on regional scales and differences in the fraction of explained variation in MODIS-NPP between biomes were large, and a combination of NDVI metrics is recommended for global studies. We have found an increasing difference between trends in climatic drivers and observed NDVI for large parts of the globe. Our findings suggest that future scenarios must consider impacts of constraints on plant growth such as extremes in weather and nutrient availability to predict changes in NPP and CO_2_ sequestration capacity.

## Introduction

Quantification of trends in ecosystem productivity is essential to understand observed and expected responses to environmental changes. Recent easing of climatic constraints have increased global vegetation productivity [1], although clear regional differences are present with both stronger positive but also negative changes in net primary production (see reproduced figure in Dent et al. [2]. For the northern latitudes, warming during the photosynthetic active period resulted in an increase in photosynthetic activity [3] and productivity [4]. Increasing atmospheric CO_2_ concentration is another contributing factor to increased productivity, estimated to be about equally important as climate [5]. Increases in productivity are limited in areas with nutrient [6] or water availability constraints and increasing drought frequency [7,8].

Studies on climate records covering long time-series with multiple decades indicate that trends are rarely monotonic, but include phases with different trends [9–11]. For vegetated areas that strongly respond to e.g. climatic drivers, short term fluctuation [12] and thus transitions in trends are to be expected in long time-series of vegetation indices, such as the Normalised Difference Vegetation Index (NDVI), as they are directly related to productivity [13,14]. Various authors have identified trend breaks in observed (wheat) yields at global and regional scales, with yields reaching a plateau in recent years after a long period of increasing yields [15–19]. These transitions have been observed in GIMMS3g NDVI for the 1982–2008 period [20]. In all areas with a significant trend in vegetation activity (15% of the global land area), trend transitions were more common (74%) than monotonic trends (26%), trend breaks and a change from greening to browning occurred more frequently in the 2000s than in the 1980s [20].

Methodological choices affect conclusions about trends in specific regions. For example in the Sahel zone, the fraction of pixels with significant positive or negative trends depends on the method used to determine a seasonal or annual value [21] and the definition of the time-period that best reflects a season [22]. A wide range of choices have been used including: annual NDVI sums [23]; sums of months around the peak of season [22]; maximum LAI values [24]; residual trends after regression with rainfall amounts based on seasonal sums for dryland areas [21,25]; or integrals based on variable start and end points (either annual or growing season) determined from phenology [26,27] or soil thawing [3]. Also the selected trend detection method can make an important difference. Compared to a simple linear regression, fitting polynomials can accommodate gradual changes [10,28] under the assumption that residuals are randomly distributed and only include “white noise” [10]. The BFAST approach is specifically designed to determine discontinuities in remote sensing datasets [29].

Explanation of observed trends in NDVI datasets is notoriously difficult as trends in coarse spatial resolution datasets such as GIMMS3g-NDVI are difficult to verify [30]. Attempts to attribute observed trends derived from the GIMMS3g-NDVI dataset to e.g. land degradation resulted in controversy about methods and interpretation of trends. See for example comments of Wessels [31] on work of Bai et al. [23] and the response of Dent et al. [2]. Extreme events such as droughts clearly influence ecosystem responses [32], further complicating the interpretation. Only few studies validated NDVI-trends with field observations of changes in biomass, for obvious reasons. Dardel et al. [22] found that two-monthly mean NDVI were moderately strong related to herbaceous mass, although changes in vegetation composition can have a strong influence [33].

A better understanding of the influence of climatic, biophysical and human induced drivers of change is needed to better predict the vegetation response in scenarios for the future. The occurrence of a negative trend in NDVI time-series with a positive trend in climatic drivers indicate a potential decoupled vegetation response. A full understanding of this potential decoupling is essential. Firstly, it will affect our estimation of the amount of terrestrial carbon sequestration under future climate scenarios [34]. Secondly, it enables a better understanding of observed ecosystem productivity with methodologies based on vegetation indices [35–37]. Thirdly, the human influence on ecosystems needs to be better understood to initiate relevant and targeted interventions. There is a considerable component of spatially correlated variation in NDVI datasets that is not related to temperature or rainfall, but that may be linked to land-use [28] or other human activity [38]. Although water availability is one of the major constraints in productivity of dryland areas, responses of vegetation indices to changes in rainfall are non-linear e.g. due to differences in rain use-efficiency [21,39]. This indicates that all climatic factors need to be weighted [40,41] or considered in combination, e.g. by using plant growth models.

This study aims to understand better a possible decoupling of observed changes in vegetation activity (as an approximation for biomass productivity) from an expected change derived from models responding to climatic drivers only. To this end, global trends in the GIMMS3g NDVI dataset covering the years 1982 to 2010 were compared with trends in modeled water-limited biomass production. The objective was to map regions where the vegetation response was decoupled from the climatic drivers and showed a negative trend in greening combined with a positive trend in modeled water-limited biomass production, indicating a decreasing productivity that may be associated with land use change or land degradation processes. To assess uncertainty due to methodological choices, trends in NDVI metrics derived from the GIMMS3g NDVI dataset (including seasonal sums, seasonal integrals and maximum NDVI), linear and segmented trend detection methods and correlations of spatial variation with MODIS-NPP and modeled water-limited biomass production were compared.

## Materials and Methods

### Remote sensing datasets

A 29 year GIMMS3g dataset derived from the series of AVHRR sensors was provided with bimonthly NDVI from 1982 up to and including 2010 [42]. The AVHRR channel 1 and 2 data, used to compute the GIMMS3g NDVI are calibrated as suggested by Vermote and Kaufman [43], and the derived NDVI is further adjusted using the technique of Los [44]. No atmospheric correction is applied to the GIMMS data except for periods (1982–1984 and 1991–1994) with stratospheric aerosols from volcanic origins [42]. A satellite orbital drift correction is performed [45], minimizing effects of orbital drift by removing common trends between time series of solar zenith angle and NDVI. The original spatial resolution of 8 km has been resampled to 5 arcminutes. Various global datasets with annual values derived from the GIMMS3g were compared: the sum of monthly means of NDVI on an annual (calendar) basis (AS-NDVI), between April and October (AO-NDVI) including the growing season in the Northern Hemisphere; between January and June (JJ-NDVI) reflecting the growing season of monsoonal climates in the southern hemisphere; between June and December (JD-NDVI) reflecting the growing season of monsoonal climates in the northern hemisphere; the large integral of the growing season (LI-NDVI); and the annual maximum NDVI (Max-NDVI) value derived with Phenolo software, for a full description see Ivits et al. [27].

The Large Integral was computed using a Savitsky–Golay filter available in the TIMESAT software [46]. These Savitsky-Golay functions were fitted to the GIMMS3g NDVI dataset using the following parameters: a seasonal parameter of 0.5; 2 envelope iterations; an adaptation strength of 1; a Savitzky–Golay window size of 2; and amplitude of the season start and season end of 20%.

The MOD17A3 dataset [47], based on the MODIS sensor, provides estimates of the annual net primary production (NPP) with an original spatial resolution of 1 km, here referred to as MODIS-NPP. MODIS-NPP is determined using NDVI and climatic data, relates reasonably well to flux-tower measurements and shows no overall bias [7,36,47]. MODIS-NPP grids for the period 2000–2010 were resampled to 5 arcminutes, exactly matching the spatial extent of grid cells in the GIMMS3g NDVI dataset.

### Ecosystems dataset

Kier et al. [48] provided detailed maps of ecoregions and 14 biomes on a global scale. These biomes were overlayed with continents, resulting in 98 unique continent-biome combinations. Land-cover was derived from the GLC2000 dataset [49], including 18 land-cover classes.

### Modelled total biomass weight

The influence of changes in daily radiation, temperature, humidity and rainfall on productivity was evaluated with LINPAC [50], a crop growth model based on the light-use-efficiency approach [51,52]. The model has been used to assess rainfed potential (i.e. water limited) biomass production at local to global scales. LINPAC computes the annual total biomass weight (TBW) produced for annual and perennial vegetation and is calibrated and validated using datasets of four perennial plant species from seven continents [53], and is also used in a comparative study for calculating maize production at four global sites [54]. The start and end of a growing season was determined by temperature and soil moisture conditions. In the calculations for this study the effects of changing CO_2_ concentrations during three decades up to 2010 have not been taken into account.

Climate data covering the years 1981–2010 were derived from the CRUTS3.10 monthly 30 arcminute resolution gridded dataset, available for each year [55,56], with weather variables recalculated to daily values as input for the crop model. Daily rainfall was computed by a random generator in combination with the number of rainy days per month to allow days without rain. For a more detailed description and references see [57]. Crop water availability is determined using a simple soil water module including one soil compartment, considering run-off, evapotranspiration and water percolation beyond rooting depth. Thickness of the soil compartment equals rooting depth up to a maximum as determined by soil limitations or maximum rooting depth of the plant species. Soil information was derived from the ISRIC-WISE database for soil properties [58], and the digital soil map of the world with a 5 arcminute resolution [59].

In this work, we quantify the influence of changes in climate on total biomass weight (TBW) of the vegetation, allowing some error in the absolute value for biomass production for a particular year and location. For each pixel and year, TBW for arable land use was determined using model parameters for a spring wheat (temperate) or a grain maize (tropical) crop. For other land uses the TBW was determined using Miscanthus (*Miscanthus* spp, a C4 species), with parameters adapted to allow growth in cold or short-season climates. A sensitivity analysis using Reed canary grass (*Phalaris arundinacea*) as C3 perennial species instead of Miscanthus showed only a minor effect on trend values [57]. The combined TBW including all land uses was calculated per pixel based on weights for the relative area of crop land and other land-use as determined by Erb et al. [60]. Ultimately, the global TBW map was produced at a spatial resolution of 5 arcminutes for the years 1981 up to and including 2010.

### Comparison of trends

Trends were estimated with a simple linear regression (SLR), a piecewise regression (PWR) and a Thiel-Sen estimator for slopes, the latter based on the median of slopes from all pair-wise combinations. The Thiel-Sen medium trend estimator is particularly useful when there are extreme values in the data-series [61]. For this, the MATLAB® software package was used, and the function Thiel_Sen_Regress was downloaded from Matlab central (http://www.mathworks.se/matlabcentral/). In a broader context, sophisticated trend analyses have been proposed [9–11]. However, the assumption that residuals are randomly distributed, so-called “white noise”, needs to be assessed. Considering the large amount of pixels, a visual pixel-by-pixel evaluation of residuals as recommended is impractical. Also, detection of a large number of different trends in a time-series, as possible in e.g. the BFAST approach [29], each covering a limited number of years is not meaningful to understand responses to long-term processes as these are strongly influenced by cyclic weather patterns. Therefore, a trend estimate should preferably cover at least one full El Nino/La Nina cycle of 4–7 years to identify long term changes rather than responses to short-term climatic variations.

In a piece-wise regression, the number of segments in the PWR can vary, but for this study only two segments were used. This was done in order to restrict the influence of events with a relative short period of influence on landscape greenness. Therefore, each segment covered at least 6 full years (equivalent to one full El Nino/La Nina cycle), i.e. the year of the breakpoint was restricted to the interval between 1 January 1988 and 31 December 2004. Events that occur within a time-series can gradually change the NDVI or cause an abrupt change. For example widespread damaging fires, logging of trees and/or severe droughts all cause a sudden but strong change in annual NDVI values and a discontinuous time-series. A PWR can accommodate this by fitting segments that are discontinuous, i.e. end points of a fitted regression line within a segment are not required to be starting points of the next segment. In this work we are more interested in gradual changes associated with long-term processes. Therefore, trends in the PWR segments were allowed to change in direction but two sequential segments were forced to pass through the same breakpoint, i.e. creating a broken stick where the two parts are still connected. This means that a single event in a stable (e.g. evergreen forest) environment, such as a damaging widespread fire with a recovery within a few years, does not trigger the start of a new segment. Such an event will affect overall average greenness, but will not strongly affect the overall trend. However, an increasing frequency or fires affecting the ability of the landscape to recover to its previous greenness or a decreasing frequency allowing the landscape to better recover will have an effect on the overall trend.

The PWR breakpoint was estimated by splitting the time-series iteratively in two segments. This process was repeated using first relatively large and then refined steps to allow optimal positioning of the breakpoint within a year. Slope values of a segment was set to zero if not different from 0 (t-test, with p < 0.1). The optimum position of the breakpoint within the time series was determined by maximizing the coefficient of determination (CD) of the PWR including both segments. A segmented regression is only meaningful when truly better than simple linear regression as it needs to compensate for the loss of one degree of freedom. This means that for our shortest time-series (1983–2010) with 28 observations, the PWR-R^2^ needs to be at least 0.034 larger than the SLR-R^2^ to compensate, i.e. for a model with a SLR-R^2^ value of 0.1 the adjusted R^2^ for PWR is also larger than the adjusted R^2^ for SLR. To remain conservative, a pixel was therefore only counted as segmented when the CD was at least 0.1 better than the SLR-R^2^ value.

A composite map was created combining PWR trend estimates for segmented pixels and SLR trends for all other land pixels. When the CD of the PWR or the R^2^ of the SLR was below 0.1, the trend values of both segments were considered insignificant and set to 0. All trend values were converted to relative values by expressing them as a percentage of the mean to enable direct comparisons between areas with low and high NDVI values. Relative values were computed as either SLR-trends (and Thiel-Sen median trends) divided by the overall mean including all years or as PWR-trend for the first or second segment divided by the mean for that specific segment respectively.

Trend values are not reliable for areas with a large bare ground fraction or many years without vegetative growth. Others have e.g. used 0.02 standard deviations in NDVI to mask out these areas [21], based on the assumption that bare areas do not vary in annual NDVI. We found that productive areas with evergreen vegetation were also occasionally below this threshold. To this end, we have defined an upper NDVI threshold of 0.3 for bare areas based on the mean AS-NDVI over the full time-series and combined this with a threshold of 0.02 standard deviations to mask bare areas, this mask was used for the figure comparing trends and breakpoints for the three metrics only.

### Comparison of annual measures of productivity

The NDVI metrics derived from the GIMMS3g dataset were related to the MODIS-NPP estimates. For each NDVI metric, the mean was determined per pixel for the years that overlapped with the MODIS-NPP dataset (2001–2010). Means for these same years were also determined for the MODIS-NPP dataset. Pixels without TBW (due to unproductive years) or NDVI (e.g. no season determined in TIMESAT) values in more than half of the years in the time-series were excluded. Simple second-order polynomial relationships between means of NDVI per pixel as independent and means of MODIS-NPP per pixel as dependent variables were established. Firstly, one single (global) relationship including all land pixels was fitted. Secondly, continent-biome stratifications were created and separate relationships were fitted for all land pixels within each continent-biome combination. All residuals and all fitted values from these continent-biome specific relationships were combined. Finally, the overall fraction of explained variation (R^2^) and root mean squared error (RMSE) values were computed using these combinations. The same was done for stratifications based on land-cover-continent combinations.

Using temporal averages of pixel values in a similar manner, NDVI metrics were also related to TBW values, however overlapping years in these datasets were 1982–2010. This provided statistics to quantify differences in NDVI metrics and influence of stratification (using biomes or land cover) on the accuracy of relationships between NDVI metrics and MODIS-NPP or TBW.

### Determining decoupled vegetation response to climatic drivers of growth

In the comparisons between TBW and NDVI trends only productive areas are considered, defined here as the area including all pixels where a TBW value was calculated in at least half of the years in the time-series. Trends in NDVI metrics were considered ***decoupled*** from its climatic drivers when the direction of the trend in NDVI was negative, despite a positive TBW trend. Pixels with a positive NDVI trend and a neutral (including all pixels with a weak trend and SLR-R^2^ or PWR-CD < 0.1) or negative TBW trend are referred to as ***diverging***, to differentiate these greening areas from browning areas. Pixels showing no or a very weak (SLR-R^2^ or PWR-CD<0.1) trend in NDVI were excluded and not counted as decoupled or diverging.

Trends in TBW were most often not segmented and (for reasons of simplicity) maps were created showing classes combining either positive or negative SLR-trends in TBW with monotonic (SLR, greening or browning) and segmented NDVI trends (PWR, greening or browning in both segments; greening followed by browning; or browning followed by greening) using the composite NDVI maps as explained above. Fractions of pixels in categories with various combinations of positive and negative TBW and these NDVI trends were determined.

For latitudinal comparisons of trends in NDVI and TBW, only productive pixels with a mean NDVI (1982–2010) above 0.1 were considered. For each pixel row, latitudinal means were determined within one degree latitude.

## Results

### Comparison of trends

**Fig 1:**
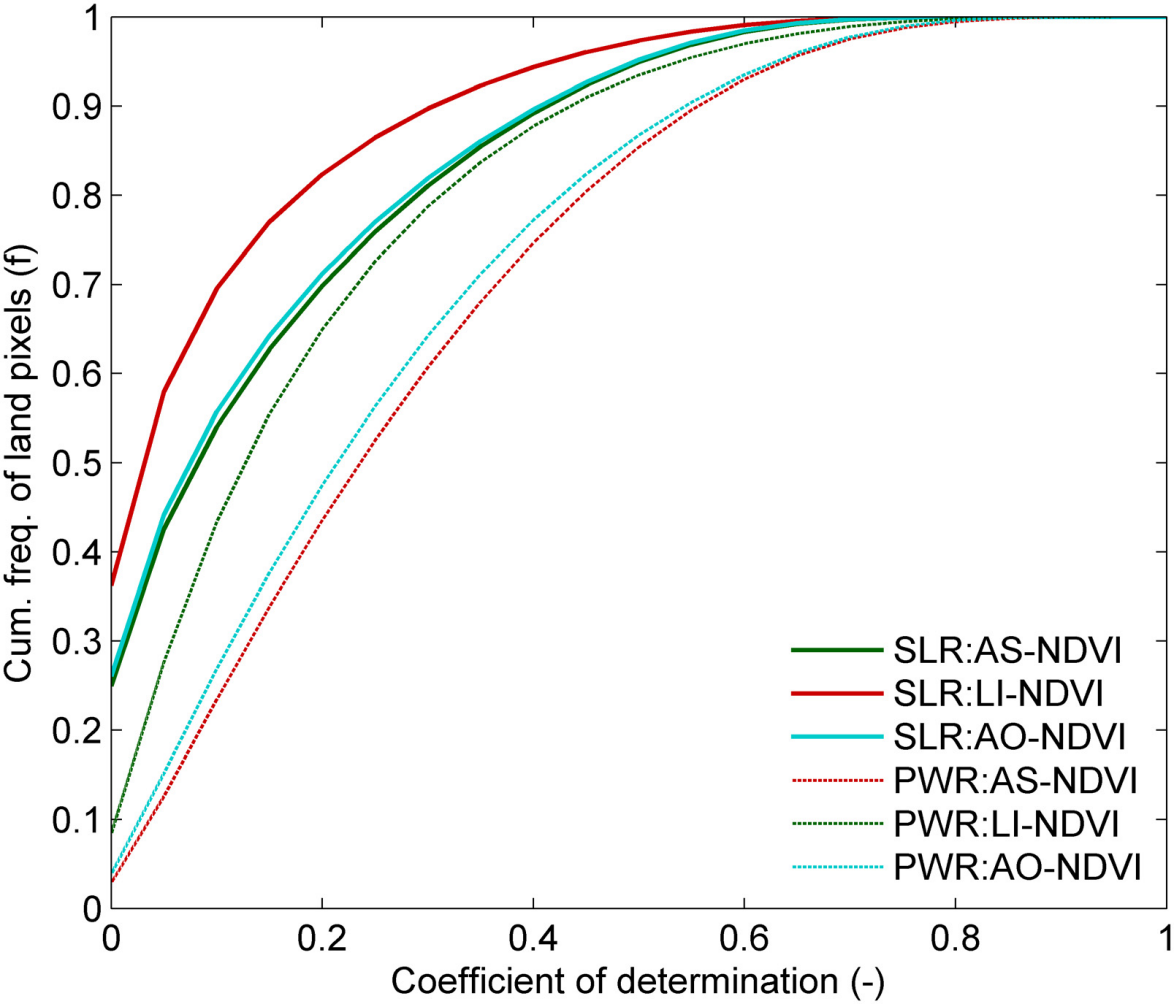
Cumulative frequency distribution of the coefficient of determination for Simple Linear Regression (SLR) and piece-wise regression (PWR) trends in land pixels for the Annual Sum (AS-NDVI), Large Integral (LI-NDVI) and April-October (AO-NDVI) GIMMS3g NDVI metrics.

Pixels with weak SLR-trends (R^2^ value < 0.3) occurred much more frequently than pixels with a moderately strong SLR-trend (R^2^ value > 0.6) (Fig 1). When compared to the SLR, the PWR increased the fraction of explained variation in the NDVI time-series, most notably in the lower R^2^-range, and PWR increased the frequency of pixels with a moderate to strong relationship. For more than 50% of the pixels in the AS-NDVI metric with weak or moderate SLR-trends (SLR-R^2^ value < 0.6), the fraction of explained variation improved with at least 0.1 when using the PWR (not shown). The frequency distributions of AS-NDVI and AO-NDVI were quite similar in each trend estimation method. The frequency of pixels with a very weak trend (SLR-R^2^ and PWR-CD < 0.1) was larger for the LI-NDVI metric when compared to the other two NDVI metrics shown in Fig 1.

**Fig 2:**
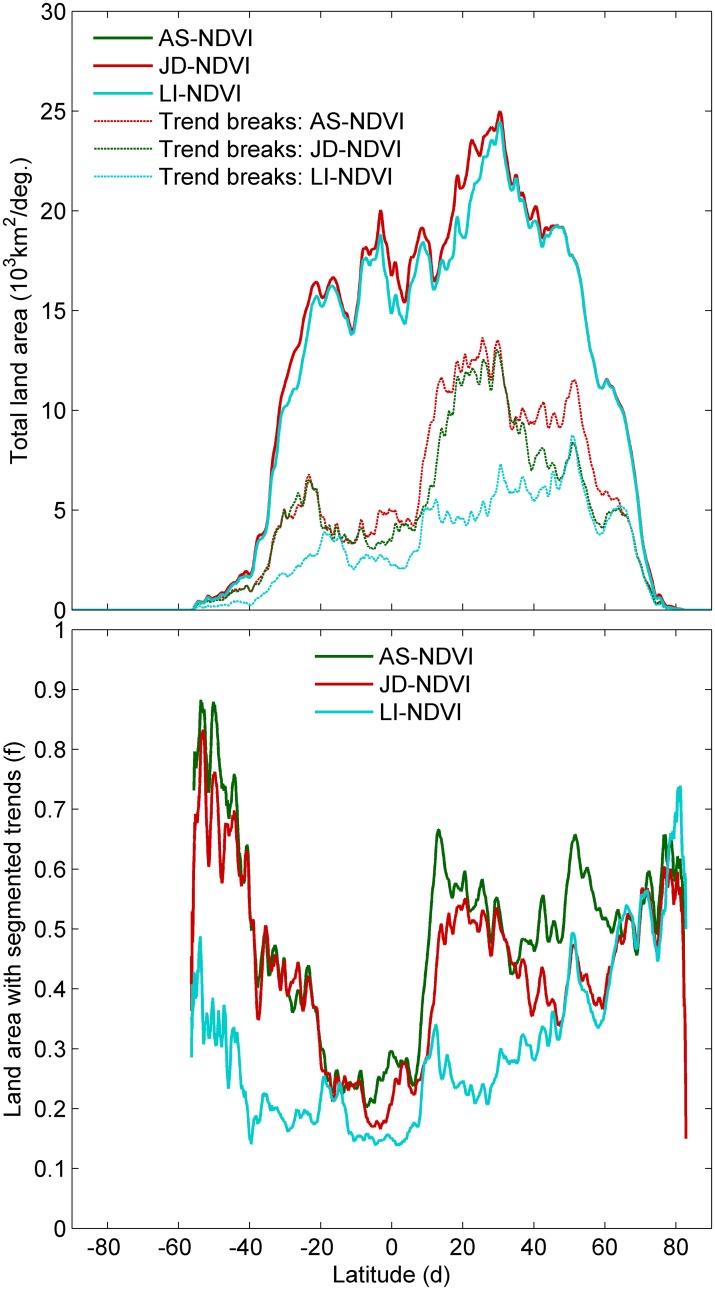
**a**, Latitudinal totals of land area and area with segmented trends for the Annual Sum (AS-NDVI), Large Integral (LI-NDVI) and April-October (AO-NDVI) GIMMS3g NDVI metrics. **b**, Fraction of land areas with segmented trends in these three datasets.

The improvement of PWR over the SLR trend method varied between the NDVI metrics (evidenced by difference in the fraction of land pixels where the PWR-CD was at least 0.1 larger than the SLR-R^2^, Table A in S1 File), in particular south of 60°N where differences between LI-NDVI and the other NDVI metrics were largest (Fig 2). The fraction of segmented pixels varied with latitude but were lowest near the equator (between 20°S and 10°N) for all datasets (Fig 2).

The TBW dataset showed mostly monotonic trends with only for 6% of the pixels a segmented trend (Table A S1 File). The SLR and Thiel-Sen trends did not differ much across the NDVI metrics, with more or less comparable proportions of pixels with a positive or negative trend (Table B in S1 File). Most of the pixels did not show strong TBW trends, the R^2^ remained below 0.1 for 72% of the productive area (i.e. a neutral trend). From all pixels in productive areas, 23% showed a positive SLR-trend in TBW indicating more favorable climatic conditions and 5% a negative SLR-trend was found for only 5% (Table B in S1 File). For TBW, the PWR did not improve the fraction of explained variation strongly, only 5% of the TBW pixels showed a segmented TBW trend (Table B in S1 File).

Globally, the difference between NDVI metrics in the number of pixels with a positive or negative SLR and Thiel-Sen trend was small (except Max-NDVI). The differences were more pronounced when looking at pixels with a segmented PWR trend, although fractions of pixels for summed NDVI metrics were similar (e.g. 14–18% with monotonic negative trending pixels and 38–40% for monotonic positive trending pixels, Table B in S1 File).

**Fig 3:**
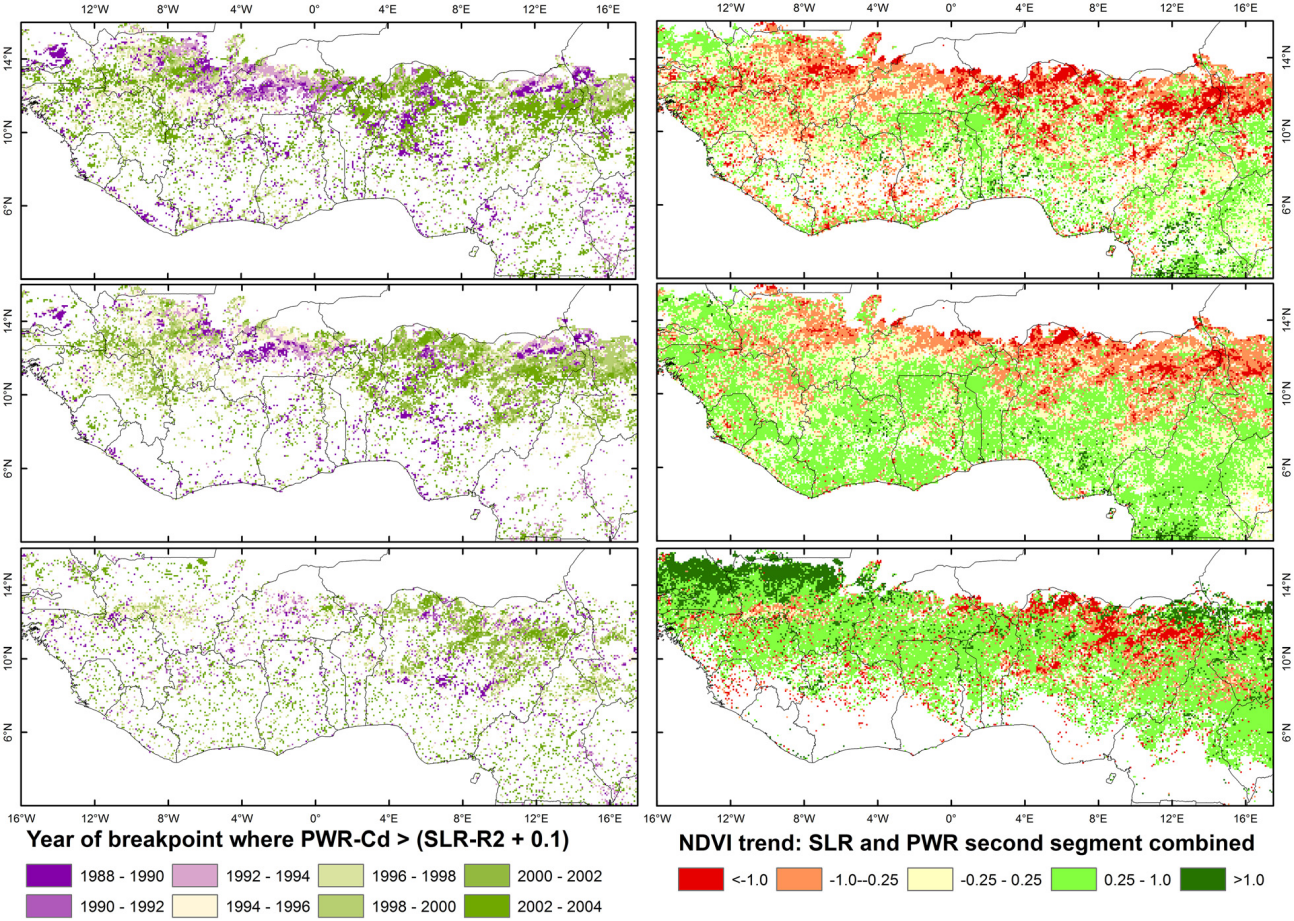
Differences in the estimated year of the breakpoint (panels a, c, e) and most recent trends (panels b, d, f) between the AO-NDVI (panels a, b), AS-NDVI (panels c, d) and LI-NDVI (panels e, f) metrics. Trends shown are the percentage change per year. Maps are combinations of SLR trends and the trend in the second segment, the PWR segment 2 trend was used when PWR explained at least 0.1 more variation than SLR. Pixels in bare areas or with a low fraction (< 0.1) of explained variation are shown as white.

The overall differences between NDVI metrics in the fraction of pixels within a specific SLR-R^2^ or PWR-CD range (see also Fig 1) were even more pronounced for biomes with evergreen vegetation, where the frequency of low CD values was much higher for LI-NDVI than for the other two metrics (compare legend numbers 1–4 in S1a–S1c Fig. a-). The differences between metrics resulted in distinct differences in estimated regional trend patterns (Fig 3). The number of unmasked pixels with a CD or R^2^ value above 0.1 were much more frequent for the AS-NDVI than AO-NDVI or LI-NDVI, in particular in the south-western areas of West Africa where evergreen vegetation occurs frequently. The percentage of annual change (e.g. Senegal) and the direction of the trends (e.g. western Mali) also differed between the NDVI metrics for particular areas. Spatially, there was more similarity and less scatter in the estimated year of the breakpoint for the AO-NDVI and AS-NDVI metrics than for the LI-NDVI metric (Fig 3).

### Comparison of annual measures of productivity

The spatial variation in mean NPP within the MOD17A3 dataset that can be explained varied between NDVI metrics (Table 1). Separate relationships for each biome or land-use type improved the fraction of explained variation and reduced RMSE values for all NDVI metrics (R^2^ of 0.72–0.80) and for TBW (R^2^ of 0.74–0.75) when compared with a single relationship (Table 1). Differences between land-use and biome specific relationships were relatively small. However, there were clear differences in the relationships across continent-biome combinations (Table 2) and land cover types. The differences in explained NPP variation between biomes or land cover types were large, e.g. for AS NDVI ranging from 22–74% with respect to biomes (Table 2) and from 12–63% for land-cover types (data not shown).

**Table 1:** Descriptive statistics for second order polynomial relationships between various metrics derived from GIMMS3G NDVI-metrics and NPP (g C m^-2^ yr^-1^ MOD17A3). Comparisons of one generic and biome/land cover (LC) specific relationships per continent.

Metric	Global			Biome specific		LC specific	
	N	R^2^	RMSE	R^2^	RMSE	R^2^	RMSE
AS-NDVI	1716630	0.72	178.2	0.79	154.9	0.79	154.7
LI-NDVI	1714527	0.69	187.3	0.77	161.0	0.78	160.4
AO-NDVI	1716624	0.56	224.0	0.76	167.1	0.77	163.5
JJ-NDVI	1716629	0.70	186.8	0.80	153.1	0.79	155.5
JD-NDVI	1716630	0.63	207.6	0.75	169.4	0.77	161.5
Max-NDVI	1716535	0.35	274.4	0.73	177.6	0.72	179.7
TBW	1559462	0.63	204.9	0.74	172.6	0.75	169.8

**Table 2:** Means and ranges of R^2^ values across continents for (quadratic) relationships between NDVI and MODIS-NPP per biome. Relationships were developed for each continent separately, shown here for the annual sum (AS), large seasonal integral (LI) and April-October (AO) NDVI metrics. For each biome, the overall best performing GIMMS3g NDVI metric (including January-June, JJ) is listed.

Biome	AS-NDVI		LI-NDVI		AO-NDVI		Best
	Mean	Range	Mean	Range	Mean	Range	
Tropical & subtropical moist broadleaf forests	0.22	0.03–0.45	0.16	0.02–0.35	0.21	0.04–0.49	AS
Tropical & subtropical dry broadleaf forests	0.38	0.17–0.68	0.32	0.10–0.59	0.42	0.23–0.72	AO
Tropical & subtropical coniferous forests	0.53	0.45–0.61	0.47	0.41–0.53	0.32	0.21–0.42	AS
Temperate broadleaf & mixed forests	0.54	0.24–0.73	0.42	0.05–0.60	0.45	0.19–0.73	AS
Temperate conifer forests	0.66	0.57–0.82	0.61	0.52–0.78	0.57	0.32–0.79	JJ
Boreal forests/taiga	0.72	0.69–0.77	0.71	0.68–0.77	0.75	0.72–0.79	AO
Tropical & subtropical grasslands	0.52	0.18–0.73	0.47	0.11–0.68	0.48	0.20–0.72	JJ
Temperate grasslands	0.69	0.47–0.83	0.66	0.50–0.81	0.74	0.60–0.83	AO
Flooded grasslands & savannas	0.41	0.11–0.66	0.29	0.02–0.71	0.31	0.05–0.75	AS
Montane grasslands & shrublands	0.67	0.47–0.75	0.59	0.19–0.75	0.63	0.45–0.77	AS
Tundra	0.74	0.66–0.85	0.73	0.65–0.85	0.74	0.64–0.87	JJ
Mediterranean forests	0.66	0.47–0.91	0.58	0.34–0.86	0.60	0.42–0.89	AS
Deserts & xeric shrublands	0.46	0.13–0.84	0.42	0.10–0.83	0.46	0.18–0.81	AS
Mangroves	0.33	0.15–0.63	0.28	0.18–0.53	0.30	0.15–0.63	JJ

On a global scale, spatial variation in NDVI correlated strongly to spatial variation in TBW (Table C in S1 File). Within biomes, between 46–67% of variation in NPP and 29–47% of variation in TBW was explained, when spatially combining the best NDVI metric per continent-biome (Table D in S1 File).

There were clear differences between biomes (Table 2) and continents (Table D in S1 File) in the explained fraction of variation when comparing continent-biome combinations. Relationships were in general weak for tropical and subtropical moist broadleaf forest and strongest for biomes in more Northern areas. Moderately strong relationships were found between NDVI metrics and MODIS-NPP for most biome-continent combinations, but the best performing NDVI metric varied per biome (Table 2).

### Determining decoupled vegetation response to climatic determinants of growth

In total, 76% of all land pixels were productive, defined in this study as pixels where a TBW value was determined in more than half of the years. Between 17–36% of all pixels in these productive areas showed a decoupled vegetation response with a negative NDVI trend combined with a positive or neutral TBW trend (Table 3). However, this occurred on all continents in small areas, for example in north-east China (S2 Fig) or between the center and north of Argentina (S3 Fig). This area in Argentina borders to a wider region in South America with negative trends in NDVI metrics, although to the north of Argentina this negative trend is combined with a negative trend in TBW indicating worsening climatic conditions (e.g. Paraguay and the far north of Argentina). In Africa, improving climatic conditions with positive TBW trends combined with negative NDVI trends occurred in a relatively small area in the north of Morocco (S4 Fig) and in a wider area between 10 and 20°S, in particular in Angola, Tanzania, Zambia and Mozambique (S5 Fig), and on Madagascar.

**Table 3:** Proportions of productive pixels (with a TBW value calculated for more than half of the years) with decoupled, diverging or following or trends. Decoupled trends combine a negative NDVI trend with a positive or neutral TBW trend, diverging trends combining a positive NDVI trend with a negative TBW trend, and pixels with following trends have NDVI trends in line with TBW trends. A proportion of 0.23, 0.72 and 0.05 of all productive pixels show positive, neutral or negative TBW trends respectively.

Metric	Decoupled		Diverging	Following	
	TBW+	TBW0	TBW-	TBW+	TBW-
AS-NDVI	0.08	0.27	0.02	0.11	0.02
LI-NDVI	0.06	0.20	0.01	0.10	0.02
AO-NDVI	0.07	0.24	0.02	0.11	0.02
JJ-NDVI	0.07	0.23	0.02	0.11	0.02
JD-NDVI	0.08	0.28	0.02	0.09	0.02
Max-NDVI	0.04	0.13	0.02	0.10	0.01

From all productive pixels with a positive TBW trend (23%, thus excluding the pixels without a TBW trend) 17–33% were decoupled, equivalent to 4–8% of all pixels in productive areas (Table 3). This decoupled trend, mostly with a negative trend in recent times, occurred in wider areas in the Sahel zone and around 50 degrees latitude in Europe and Asia (S2 and S4 Figs, please note that the observed patterns differ between metrics). For the remaining pixels with a positive TBW trend, a fraction of 38–47% showed positive trends, equivalent to 9–11% of all productive pixels (Table 3). The areas showing a greening trend in line with climatic drivers after an declining period (see e.g. S3 and S5 Figs: central-south Angola, Ethiopia, Mexico and western edge of Brazil), may indicate some recovery.

From all productive pixels, 5% showed a negative TBW trend. From these pixels, 30–36% were diverging, indicating recovery, equivalent to 1–2% of all pixels in productive areas (Table 3). This was observed near the Loess plateau in northeast China (S1 Fig, both LI-NDVI and AS-NDVI) and the agricultural areas in northern India (S2 Fig, only AS-NDVI) and southwest Australia (S7 Fig).

**Fig 4:**
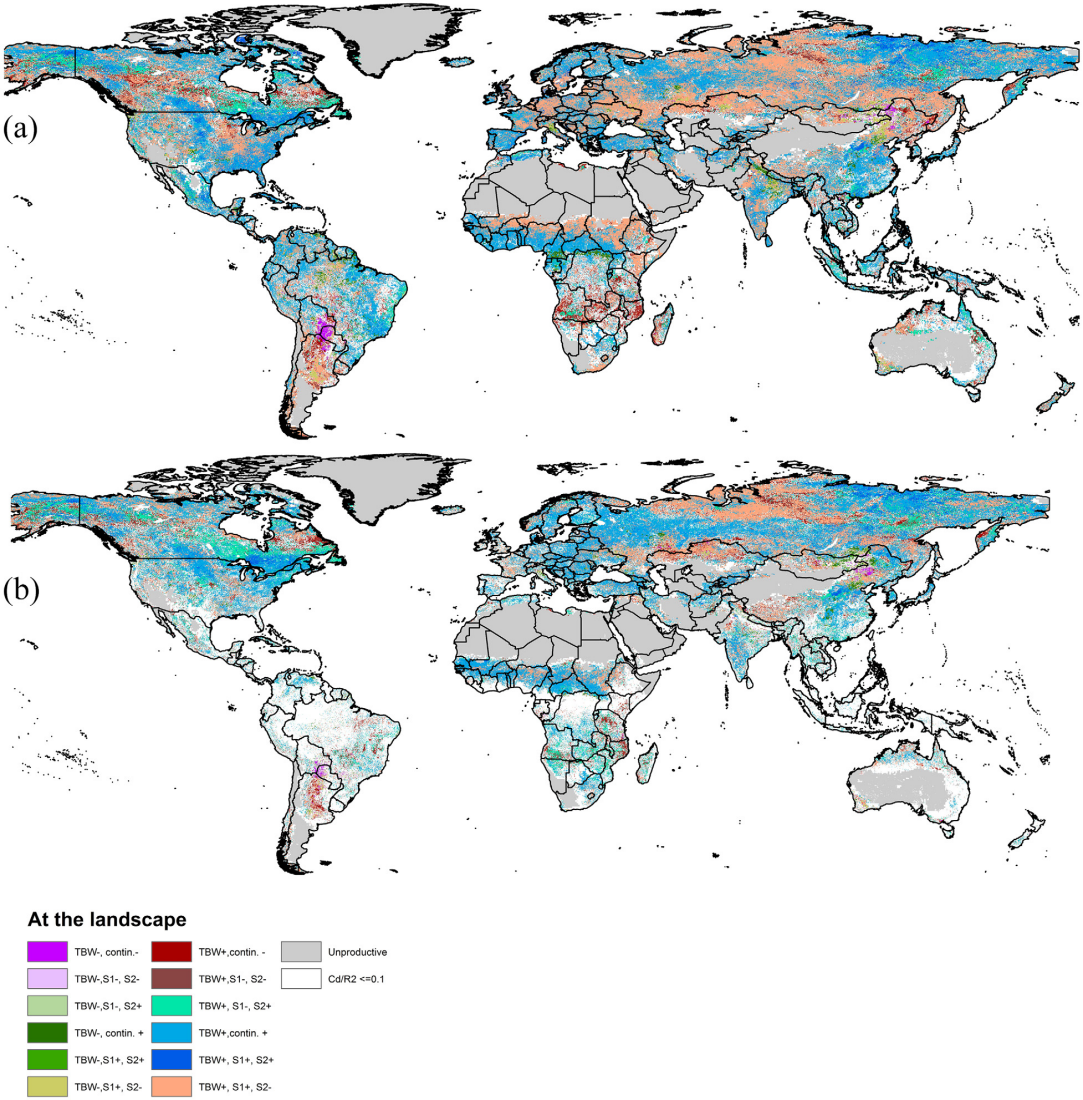
**a**, Global map showing combinations of SLR-trends in TBW and PWR-trends in the two segments (S1 and S2 for the two segments) for the AS-NDVI metric. The “+” sign in the legend indicates a neutral or positive trend, a “–”sign a negative trend. For white areas, the temporal trend explained less than 10% of the observed variation in AS-NDVI or TBW. A continuing trend indicates that PWR did not improve upon a SLR trend and the later trend value was used; **(b)**: as in (**a)** but now for the LI-NDVI metric. See supplementary materials for more detailed figures for separate regions.

The fraction of pixels with decoupled or diverging TBW and NDVI trends varied only slightly between metrics on a global scale, with exception of Max-NDVI (Table 3). However, differences between NDVI metrics were important when studying specific geographical regions, as illustrated in Fig 3 (Sahel) and Fig 4 (e.g. Belarus and Ukraine).

**Fig 5:**
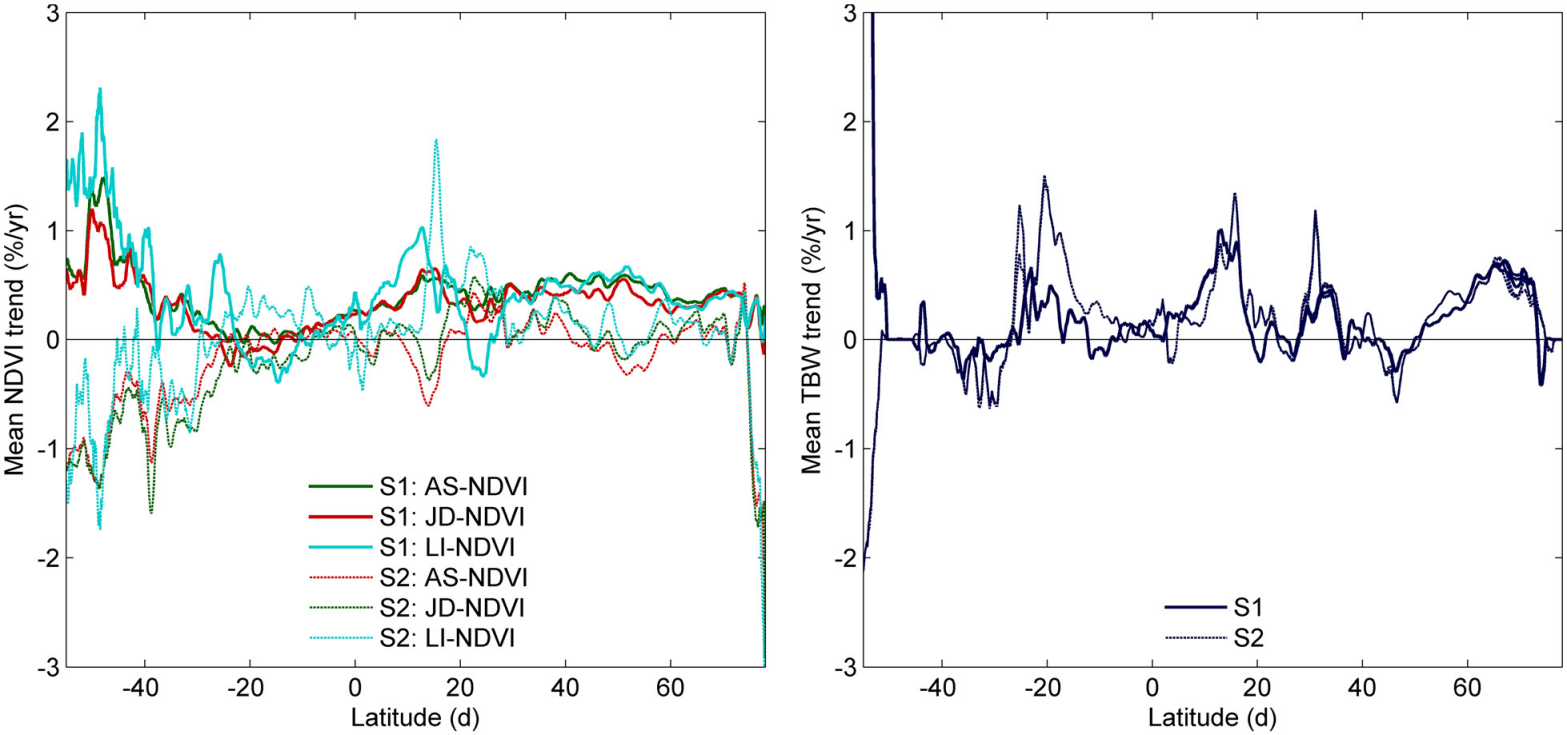
**a**, Latitudinal means of NDVI metrics based on AS-NDVI and AO-NDVI for the years 1982–2010 (for legend see Fig 5c); **b,** mean year of breakpoint for pixels where PWR regression improved upon SLR; **c,** mean trends in three NDVI metrics for the first (S1) and second segment (S2) of PWR. Pixel trends were based on PWR where PWR improved the coefficient of determination with at least 0.1 and SLR trends otherwise; (**d)** as in (**c**) but now for TBW.

On a global scale, a decoupling between changes in vegetation activity and modelled productivity was visible for all datasets (Table 3 and compare Fig 5c and 5d). Generally, NDVI trends in the first PWR segment were positive and more neutral for the second PWR segment, whereas TBW trends remained positive in both PWR segments for most latitudes (Fig 5c and 5d). A negative TBW trend was found for latitudinal means between 25–40°S and 40–50°N. The year when a breakpoint between PWR segments was determined showed a latitudinal pattern, somewhat similar to mean NDVI values, with breakpoints identified in later years for latitudes with lower mean NDVI values (S8a and S8b Fig).

## Discussion

This work shows a clear difference in the area with monotonic and segmented trends and direction of these trends when comparing climate-constrained, modelled biomass production with observed NDVI for large parts of the globe. A decoupled NDVI-trend, with decreasing vegetation activity opposing a positive or neutral trend in climatic drivers, was observed for 17–36% of the pixels in productive areas depending on the NDVI metric used. Only a small proportion (6–9%) of these productive areas were trending downwards in NDVI over the full period from 1982–2010, and the majority of these areas showed a transition of trends, with first a positive and then a negative trend in NDVI. These findings are effected by uncertainties in the GIMMS3g dataset, as discussed elsewhere [62]. Most of the pixels with a decoupled trend had a neutral TBW trend, aligning with the overall dominance of neutral trends in TBW (72%). This could be due to the relatively short period (29 years) where impacts of changes in climatic conditions on TBW may be limited in comparison to year to year weather variation.

Observed changes in CO_2_ concentrations and temperature are major drivers of increasing landscape greenness over all latitudes, whereas the influence of changes in the amount of precipitation varies strongly on regional scales [4]. For the northern hemisphere above 50°N, strong greening kept pace with the increased temperatures when averaging over latitudes [3]. Greening rates in tropical mountains increase with altitude but greening reversed to browning in the mid-1990s at lower elevations [63]. We found that on a global scale, only 38–47% of pixels with a positive TBW were indeed greening up (as observed with NDVI), equivalent to 9–11% of all productive pixels. The large fraction of pixels with a positive (and often segmented) NDVI trend, in particular in agricultural areas, aligns with a positive trend in agricultural productivity and yield, most probably resulting from a combination of improved climatic conditions, increasing nutrient applications and soil fertility [64], rising CO_2_ concentrations and genetic improvements [65] and changes in seeding times [19]. Plateauing crop yields have been reported for various parts of the world [15–18], for time intervals comparable to our study that can be linked to biophysical constraints to crop productivity [66]. Hence, the positive and often segmented NDVI trend in the 1981–2010 period is reasonably well understood for cropland areas considering the strong relationships between yield and NDVI [67,68].

Segmented trends caused by transitions from greening to browning in non-agricultural areas have been associated with changes in rainfall patterns and temperature, resulting in larger net evapotranspiration deficits [63,69] and decreased NPP [7]. Modelled biomass integrates these effects of temperature, rainfall amounts and distribution and shifts in season lengths [50,53]. For the majority of productive areas, the trend in modelled biomass was absent or very weak (R^2^ < 0.1), about 24% showed an increase in modelled production and only 5% a decrease. Mao et al. [4] found that modelled change in landscape greenness strongly varied with latitude, increasing in wet climates between 10°S and 15°N and above 50 °N but decreasing in drier climates between 10–40 °S and 10–30 °N. We also found large differences in TBW trends between latitudes, although TBW trends were positive over a wide range of latitudes, with a negative TBW trend in latitudes from 25–40°S and 40–50°N [4]. First signs of carbon sink saturation are reported for Europe [70,71]. Higher temperatures and longer growing seasons resulted in more biomass production, whereas climate change has not resulted in an increase of the land area under drought in the northern hemisphere [72]. This in contrast to the southern hemisphere [72], where for latitudes from 25–40 °S evapotranspiration deficits are increasing for large areas in all three continents [32]. In general, rising CO_2_ concentrations increase landscape greenness [4], rendering our estimates of changes in biomass conservative as CO_2_ concentration increases that accelerate growth were not included in our study. The modelled biomass production could capture the global spatial variability in MODIS-NPP reasonably well (R^2^ values of 0.63–0.75), with similar fractions of explained variation when compared to AS-NDVI (R^2^ values of 0.72–0.79). Part of the unexplained variation may arise from spatial variation in MODIS-NDVI as TBW was calculated with only climatic variables, in contrast to MODIS-NPP that also uses MODIS-NDVI.

Decoupling between positive climatic drivers and a negative NDVI response, predominantly in the last PWR segment, may be understood when considering constraints on landscape greenness other than climate. The vegetation present within the landscape may not be able to respond to changes in climate, e.g. a drought induced productivity declines of white spruce in Alaska [73] leading to a biome shift [69]. Changes in severity and area of burning and/or fire frequency [74] may be another important component affecting NDVI trends. Nutrient constraints are prevalent in wide areas on the globe [6]. Changes in nitrogen deposition may, therefore, increase or decrease NDVI [4]. Changes in rainfall amounts and distribution influence nutrient constraints in dryland areas, e.g. Delgado-Baquerizo et al. [75] found that aridity controls N and P availability. The intensity of rainfall affects the depth of the wetted root zone, determining the amount of nutrients in the soil profile that are available for plant uptake [76]. Nutrient constraints may also result in CO_2_ acclimation [77], restricting tree stem growth response to increased CO_2_ availability in temperate [78] and tropical forests [79], evidenced by a deteriorating mineral nutrition of trees in Europe [80].

Human influences are more difficult to assess. Land use change (e.g. due to increasing cropping areas) may result in a positive [81] or a negative trend in NDVI, the latter might occur when mean NDVI for natural vegetation is higher than for crops, such as in e.g. Argentina [82]. In this area, changes in the observed start of season indicate conversion of natural vegetation into crops [83]. However, on a global scale land use change is not expected to affect NDVI as strongly as the changes in climate or CO_2_ [4]. Results of Seaquist et al. [84] suggest that “demographic and agricultural pressures in the Sahel are unable to account for differences between simulated and observed vegetation dynamics, even for the most densely populated areas”. Firstly, the human influence may be masked by changes in natural vegetation. It is suggested that native vegetation in the Sahel has been in a long-term recovery period after the 1970–1980 drought [85], coinciding with increasing rain-use efficiency [21,86]. These increasing rainfall use efficiencies may be understood when considering limited access to nutrients without water sufficiently wetting the soil during drought periods [76], resulting in an increased nutrient availability in wetter periods. Secondly, soil degradation may lead to land abandonment and eventually lead to an increase in NDVI. All NDVI metrics showed greening in the peanut-basin of Senegal. This area has undergone land-abandonment, with agricultural land-use dropping from 80% to 67% between 1980 and 2000, resulting in increasing tree cover [87], and a higher NDVI as the natural vegetation has a stronger NDVI amplitude than crops [88]. Secondly, soil fertility decline on agricultural farms may be most visible on a scale much smaller than the course spatial resolution of the GIMMS3g dataset as according to Giller et al. [89] “differences in soil fertility between fields within a single farm may be as wide as those found between agroecological zones”.

Differences due to methodological choices strongly affected trend estimates and the fraction of pixels with a positive or negative trend in specific regions strongly differed between NDVI metrics. For tropical and sub-tropical broadleaf forest biomes, the explained fractions of variation in NPP or TBW were small for a few continents (e.g. North and South America). For these biomes the response of GIMMS3g-NDVI differed strongly from MODIS [90]. The NDVI is likely not the best index for these biomes with large amounts of green biomass due to saturation, and different results are obtained when other indices (e.g. the enhanced vegetation index, EVI) are used [13,14]. However, MODIS-EVI underestimates GPP in African landscapes [37], suggesting that selection of a vegetation index is not trivial. The differences between NDVI metrics indicate that regional assessments based on trends in a single NDVI metric, derived from e.g. the GIMMS3g dataset, needs to be interpreted with the highest caution as these trends are affected by a large degree of uncertainty. For global studies, it is critical to be able to select the appropriate NDVI metric for the purpose, but this is not a trivial choice and influences the trend estimate and direction of trends on regional scales. Further study is needed to better understand which NDVI-metrics are best suited to particular biomes or eco-regions. In the absence of metrics with long-term biomass observations suited for comparisons with GIMMS3g data, a comparison with independent data (e.g. MODIS or other sensors) is recommended [62].

Discontinuous trends in GIMMS3g time series result in differences between observed trends for particular epochs. NDVI trends were segmented for 32–48% of the pixels, confirming findings of others [28,91] and in line with the type of trends observed in climatic datasets [9–11]. The frequent presence of discontinuous trends can explain differences in trend estimates when a slightly different time-period is used [22]. This highlights the importance of these long-term datasets, enabling to differentiate between external factors influencing trends and long-term cyclic patterns in landscape greenness. The presence of discontinuous trends also indicates that there is uncertainty about the linearity and on the duration of a particular trend, requiring extra caution when extrapolating trends into the future. The approach presented here provides an improvement of our understanding of the vegetation responses to changes in climate and provides better means to monitor and assess the human influence on ecosystem productivity.

The observed segmented and often decoupled trends in ecosystem productivity opposing climatic drivers may have been caused by a combination of natural and human factors including land use change, declining soil fertility, nutrient limitations, biome shifts and effects of fire, but disentangling these remains challenging. This work stresses the importance of a better understanding of current limitations to plant growth and suggests that future scenarios must consider the influence of droughts and extreme weather events, nutrient availability and other constraints on predicted changes in NPP.

## Supporting Information

S1 Fig Cumulative frequency distribution of Piecewise linear regression (PWR) coefficient of determination (CD) values within biomes for the AS-NDVI (a), LI-NDVI (b) and AO-NDVI (c) metrics. Legend numbers indicate cumulative frequency distributions of the following biomes: 1 Tropical and subtropical moist broadleaf forests; 2 Tropical and subtropical dry broadleaf forests; 3 Tropical and subtropical coniferous forests; 4 Temperate broadleaf and mixed forests; 5 Temperate conifer forests; 6 Boreal forests/taiga; 7 Tropical and subtropical grasslands, savannas and shrub lands; 8 Temp. grasslands, savannas and shrub lands; 9 Flooded grasslands and savannas; 10 Montane grasslands and shrub lands; 11 Tundra; 12 Mediterranean forests, woodlands and shrub; 13 Deserts and xeric shrub lands; 14 Mangroves.
(TIF)

S2 Fig South-East Asia region. The map shows combinations of SLR-trends in TBW and PWR-trends in the two segments (S1 and S2 for the two segments) for **(a)** the AS-NDVI dataset and **(b)** the LI-NDVI dataset. The “+” sign in the legend indicates a neutral or positive trend, a “–”sign a negative trend. For white areas, the temporal trend explained less than 10% of the observed variation in AS-NDVI or TBW. A continuing trend indicates that PWR did not improve upon a SLR trend and the later trend value was used.
(TIF)

S3 Fig South and central America region. The map shows combinations of SLR-trends in TBW and PWR-trends in the two segments (S1 and S2 for the two segments) for **(a)** the AS-NDVI dataset and **(b)** the LI-NDVI dataset. The “+” sign in the legend indicates a neutral or positive trend, a “–”sign a negative trend. For white areas, the temporal trend explained less than 10% of the observed variation in AS-NDVI or TBW. A continuing trend indicates that PWR did not improve upon a SLR trend and the later trend value was used.
(TIF)

S4 Fig Wider Europe region. The map shows combinations of SLR-trends in TBW and PWR-trends in the two segments (S1 and S2 for the two segments) for **(a)** the AS-NDVI dataset and **(b)** the LI-NDVI dataset. The “+” sign in the legend indicates a neutral or positive trend, a “–”sign a negative trend. For white areas, the temporal trend explained less than 10% of the observed variation in AS-NDVI or TBW. A continuing trend indicates that PWR did not improve upon a SLR trend and the later trend value was used.
(TIF)

S5 Fig Africa. The map shows combinations of SLR-trends in TBW and PWR-trends in the two segments (S1 and S2 for the two segments) for **(a)** the AS-NDVI dataset and **(b)** the LI-NDVI dataset. The “+” sign in the legend indicates a neutral or positive trend, a “–”sign a negative trend. For white areas, the temporal trend explained less than 10% of the observed variation in AS-NDVI or TBW. A continuing trend indicates that PWR did not improve upon a SLR trend and the later trend value was used.
(TIF)

S6 Fig North America. The map shows combinations of SLR-trends in TBW and PWR-trends in the two segments (S1 and S2 for the two segments) for **(a)** the AS-NDVI dataset and **(b)** the LI-NDVI dataset. The “+” sign in the legend indicates a neutral or positive trend, a “–”sign a negative trend. For white areas, the temporal trend explained less than 10% of the observed variation in AS-NDVI or TBW. A continuing trend indicates that PWR did not improve upon a SLR trend and the later trend value was used.
(TIF)

S7 Fig Australia and surrounding region. The map shows combinations of SLR-trends in TBW and PWR-trends in the two segments (S1 and S2 for the two segments) for **(a)** the AS-NDVI dataset and **(b)** the LI-NDVI dataset. The “+” sign in the legend indicates a neutral or positive trend, a “–”sign a negative trend. For white areas, the temporal trend explained less than 10% of the observed variation in AS-NDVI or TBW. A continuing trend indicates that PWR did not improve upon a SLR trend and the later trend value was used.
(TIF)

S8 Fig **(a)** Latitudinal means of NDVI metrics based on AS-NDVI and AO-NDVI for the years 1982–2010; **(b)** Mean year of breakpoint for pixels where PWR regression improved upon SLR; mean trends in **(c)** three NDVI metrics and **(d)** TBW for the first (S1) and second segment (S2) of PWR. Pixel trends were based on PWR where PWR improved the coefficient of determination with at least 0.1 and SLR trends otherwise.
(TIF)

S1 File Supplementary material including tables. (PDF)
