# Supplementary figures and images for: Trends in Global Vegetation Activity and Climatic Drivers Indicate a Decoupled Response to Climate Change
Source: PLoS One. 2015 Oct 14;10(10):e0138013. doi: 10.1371/journal.pone.0138013 (PMC4605512; doi:10.1371/journal.pone.0138013)

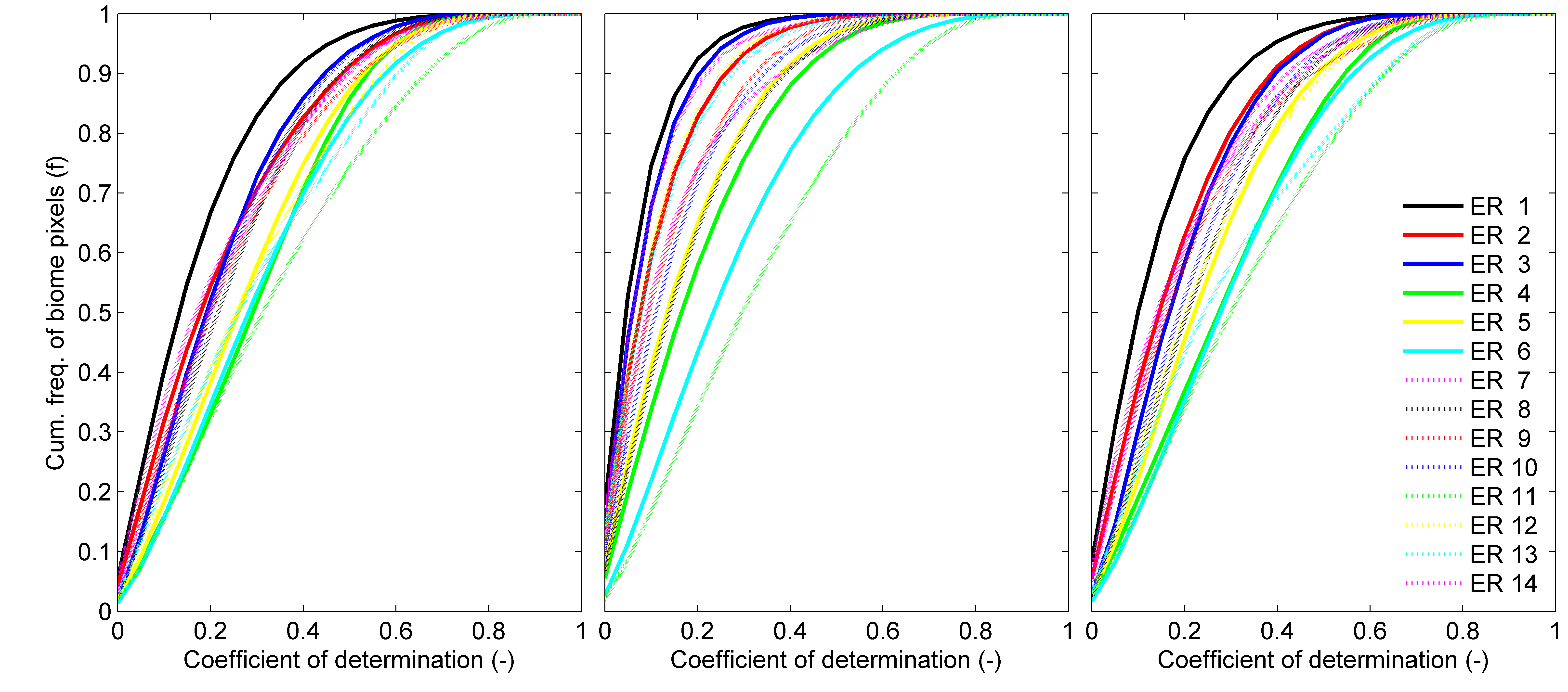

Supplement: S1 Fig — Legend numbers indicate cumulative frequency distributions of the following biomes: 1 Tropical and subtropical moist broadleaf forests; 2 Tropical and subtropical dry broadleaf forests; 3 Tropical and subtropical coniferous forests; 4 Temperate broadleaf and mixed forests; 5 Temperate conifer forests; 6 Boreal forests/taiga; 7 Tropical and subtropical grasslands, savannas and shrub lands; 8 Temp. grasslands, savannas and shrub lands; 9 Flooded grasslands and savannas; 10 Montane grasslands and shrub lands; 11 Tundra; 12 Mediterranean forests, woodlands and shrub; 13 Deserts and xeric shrub lands; 14 Mangroves. (TIF) [file pone.0138013.s001.tif]

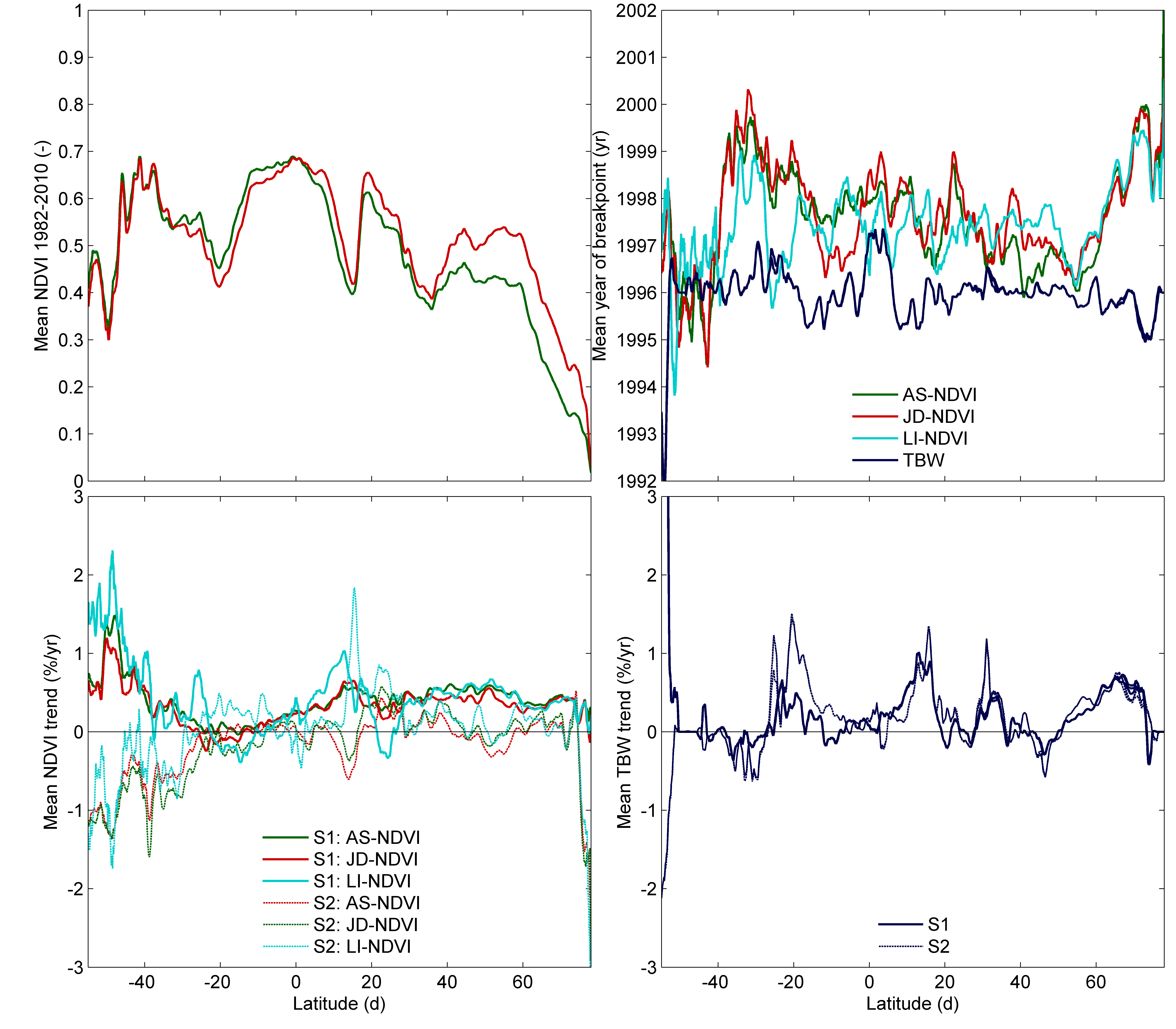

Supplement: S8 Fig — (a) Latitudinal means of NDVI metrics based on AS-NDVI and AO-NDVI for the years 1982–2010; (b) Mean year of breakpoint for pixels where PWR regression improved upon SLR; mean trends in (c) three NDVI metrics and (d) TBW for the first (S1) and second segment (S2) of PWR. Pixel trends were based on PWR where PWR improved the coefficient of determination with at least 0.1 and SLR trends otherwise. (TIF) [file pone.0138013.s008.tif]
