# Supplementary material for: Trends in Global Vegetation Activity and Climatic Drivers Indicate a Decoupled Response to Climate Change
Source: PLoS One. 2015 Oct 14;10(10):e0138013. doi: 10.1371/journal.pone.0138013 (PMC4605512; doi:10.1371/journal.pone.0138013)
Supplement: S1 File — (PDF) [file pone.0138013.s009.pdf]

SUPPLEMENTARY MATERIAL

All summed NDVI metrics had a higher fraction of segmented pixels (0.44-0.45) than the LI-NDVI (0.32) or the Max-NDVI (0.37) metrics (Table S1). The majority of the pixels with a segmented trend showed a positive change in the first segment followed by a negative change in the second segment, for all NDVI derived metrics. The maximum NDVI showed a lower fraction of pixels with segmented trends (0.37), with only a small fraction of pixels with a negative trend in the second segment (0.17), whereas more pixels showed a positive trend in both segments (0.14).

Table A. Fractions of pixels with a positive and/or negative trend in segment 1 and 2, where a piecewise linear regression improved the coefficient of determination of SLR with at least 0.1.

**Segment 1**<br>**Segment 2**	***Positive***<br>***Positive***	***Negative***<br>***Positive***	***Positive***<br>***Negative***	***Negative***<br>***Negative***	***Total***
AS-NDVI	0.05	0.08	0.33	0.02	0.48
LI-NDVI	0.03	0.10	0.19	0.01	0.32
AO-NDVI	0.05	0.08	0.29	0.02	0.44
JJ-NDVI	0.04	0.07	0.32	0.02	0.45
JD-NDVI	0.04	0.09	0.26	0.02	0.44
Max-NDVI	0.14	0.07	0.17	0.00	0.37
TBW	0.02	0.02	0.02	0.00	0.06

The proportion of pixels with a positive SLR-trend (0.62-0.72) was larger than the proportion of pixels with a negative trend (0.28-0.38), with similar proportions across the various NDVI metrics (Table B). The Thiel-Sen resulted in a total pixel proportion of 0.30-0.37 (adding up the values in the SLR positive and SLR negative rows per metric or dataset) with negative trends and 0.62-0.70 with a positive trend. The fraction of pixels with a monotonic trend, i.e. pixels where PWR did not improve on SLR, was larger for the SLR positive than for the SLR negative trend

pixels. For TBW, the Thiel-Sen method resulted in much lower proportions of pixels with a positive trend, 0.71 vs. 0.95 when compared to SLR (Table B).

Table B. Comparisons between NDVI metrics and Total modelled Biomass Weight (TBW) of the proportions of pixels with a positive and negative trend determined with a simple linear regression (SLR), Thiel-Sen median slope or Piecewise regression with two segments. The proportions for the Thiel-Sen median and the piecewise regression methods are differentiated for pixels with a negative or positive SLR slope.

**Metric/dataset**	**SLR**	**Thiel-Sen**		**Piecewise regression**[ǂ]		
**(1983-2010)**	**All**	**Pos.**	**Neg.**	**Pos.**	**Neg.**	**Monotonic**
			SLR Negative			
AS-NDVI	0.36	0.03	0.33	0.02	0.19	0.14
LI-NDVI	0.37	0.06	0.31	0.03	0.10	0.24
AO-NDVI	0.36	0.03	0.33	0.02	0.18	0.16
JJ-NDVI	0.37	0.03	0.34	0.02	0.19	0.16
JD-NDVI	0.38	0.03	0.35	0.02	0.17	0.18
Max-NDVI	0.28	0.02	0.26	0.02	0.10	0.16
TBW	0.05	0.00	0.04	0.00	0.00	0.04
			SLR Positive or zero			
AS-NDVI	0.63	0.60	0.03	0.11	0.15	0.38
LI-NDVI	0.63	0.59	0.04	0.10	0.09	0.44
AO-NDVI	0.64	0.62	0.02	0.11	0.13	0.40
JJ-NDVI	0.63	0.60	0.03	0.10	0.14	0.39
JD-NDVI	0.62	0.59	0.02	0.11	0.10	0.40
Max-NDVI	0.72	0.68	0.04	0.18	0.07	0.47
TBW	*0.95*	*0.71*	*0.24*	*0.03*	*0.02*	*0.90*

[ǂ]The Monotonic column indicates the fraction of pixels with a segmented trend where the PWR did not improve upon the SLR. The Pos. trend column indicates the total fraction of pixels with a positive trend in the second segment, including both positive and negative trends in the first segment. The Neg. trend column indicates the total fraction of pixels with a negative trend in the second segment, including both positive and negative trends in the first segment.

The RMSE values for the LI-NDVI metric was smaller, as much more pixels were excluded due to missing values (Table C). The differences between metrics were only small when looking at the biome-continent combinations, were overall the AS-NDVI showed highest R^2 values and the Max-NDVI the lowest R^2 values. Differences between continents were small, with more than 0.5 of variation explained for Africa, Asia, Australia and Europe (Table D).

Table C. R^2 values and root mean square errors (RMSE) for second order linear relationships between various GIMMS3g NDVI-metrics and LINPAC modelled water-limited total biomass weight (TBW). Comparisons of one global and biome or land use specific relationships per continent.

Metric	Global			Biome-cont.		Land use-cont.	
	N	R^2	RMSE	R^2	RMSE	R^2	RMSE
AS-NDVI	1373933	0.61	12.6	0.79	9.2	0.77	9.7
LI-NDVI	1172198	0.60	9.7	0.77	7.4	0.74	7.8
AO-NDVI	1373933	0.40	15.5	0.79	9.3	0.76	9.9
JJ-NDVI	1373922	0.59	12.9	0.78	9.3	0.76	9.9
JD-NDVI	1373933	0.50	14.2	0.78	9.5	0.76	9.8
Max-NDVI	1373732	0.16	18.5	0.75	10.1	0.70	11.0

Stronger relationships in specific metrics were found for tropical forests in Asia and Australia (R^2 of 0.47 for JJ-NDVI and 0.60 for JD-NDVI respectively) and subtropical forests in Asia and Africa (R^2 of 0.72 and 0.47 for AO-NDVI). For most other biomes, strength of relationships varied strongly between continents (Table D).

Table D. Means of R^2 values per continent for (quadratic) relationships between NDVI and MODIS-NPP for each biome. Relationships were developed for each continent separately, shown here for the annual sum (AS-NDVI), large integral (LI-NDVI) and April-October (AO-NDVI) metrics. The combined column indicates average R^2 values when metrics with the strongest relationships per biome are combined.

	NPP				TBW
Continent	AS-NDVI	LI-NDVI	AO-NDVI	Combined	Combined
World	0.53	0.47	0.49	0.58	0.39
Africa	0.58	0.54	0.48	0.67	0.47
Asia	0.61	0.58	0.55	0.64	0.44
Australia	0.59	0.44	0.56	0.63	0.33
N-America	0.46	0.40	0.43	0.49	0.40
Oceania	0.42	0.33	0.43	0.46	0.29
S-America	0.42	0.37	0.44	0.49	0.47
Europe	0.59	0.53	0.56	0.61	0.33
